# Effects of Temperature, Stoichiometric Ratio, and Crystal Orientation on the Nanoindentation Response of ZrC: A Molecular Dynamics Study

**DOI:** 10.3390/ma19122581

**Published:** 2026-06-15

**Authors:** Guiyu Liu, Hongya Zheng, Fugen Deng, Yulu Zhou, Yifang Ouyang

**Affiliations:** State Key Laboratory of Featured Metal Materials and Life-Cycle Safety for Composite Structures, Guangxi Key Laboratory for Relativistic Astrophysics, School of Physical Science and Technology, Guangxi University, Nanning 530004, China

**Keywords:** zirconium carbide, nanoindentation, molecular dynamics, mechanical properties, temperature, off-stoichiometry, anisotropy

## Abstract

The nanoindentation analysis of zirconium carbide (ZrC) has been studied through molecular dynamics simulations, focusing on various factors such as temperature, stoichiometric ratio, and crystal orientation. The findings show that as temperature increases, both the critical pop-in load and the maximum load decrease, while atomic strain, von Mises stress, and residual indentation depth increase. High temperatures facilitate the nucleation and propagation of 1/2<110> dislocations, which enhance the material’s ability to undergo plastic deformation. Both indentation hardness and Young’s modulus decrease linearly as temperature rises or the concentration of C vacancy increases. For stoichiometric ZrC, as the temperature rises from 10 K to 2100 K, the hardness decreases from 45.04 GPa to 20.36 GPa, and Young’s modulus drops from 396.28 GPa to 254.45 GPa. At 10 K, when the C/Zr ratio is reduced to 0.5, the hardness and Young modulus decrease to 25.32 GPa and 192.09 GPa, respectively. This reduction is attributed to the weakening of Zr-C bonds, which also reduces stress concentration. At elevated temperatures, the impact of C vacancies on the nanoindentation process diminishes due to the thermal softening of the substrate, which lessens the effects of vacancy-induced softening. Regarding anisotropy, Young’s modulus at room temperature decreases from 383.39 GPa on the (001) plane to 335.93 GPa on the $(1\overset{-}{1}0)$ plane, and it reduces further to 303.31 GPa on the $(1\overset{-}{1}1)$ plane; hardness shows a similar decreasing trend. This trend is primarily due to differences in slip systems, surface energies, and the angles between the plane normal and the Zr-C bond axis located directly beneath the surface atoms. Overall, these results may provide theoretical support for the processing and application of ZrC.

## 1. Introduction

Zirconium carbide (ZrC) has attracted significant attention for ultra-high-temperature applications in the nuclear and aerospace industries due to its high melting point (3530 °C), remarkable hardness (20 GPa), good thermal conductivity (40 W/(m·K)), and excellent resistance to corrosion and irradiation [1,2]. ZrC shows great potential as a coating material for advanced high-temperature reactor fuels, structural components in fusion reactors, and critical applications in propulsion systems, such as rocket engine nozzles and hypersonic vehicle wing leading edges [3,4]. For instance, in high-temperature gas-cooled reactors, ZrC is considered one of the most promising alternative ceramic materials to silicon carbide (SiC) for coating tristructural isotropic (TRISO)-coated fuel particles [5]. The ZrC layer serves as a pressure vessel for the TRISO fuel [4,6], making it essential to understand its mechanical response.

The operating temperature of ZrC in a very-high-temperature reactor ranges from 875 to 1275 K. Under extreme accident conditions, it may reach a maximum temperature of 1875 K [7,8]. The mechanical properties of ZrC can significantly deteriorate at high temperatures. In early experimental research, Baranov et al. [9] investigated the mechanical response of ZrC at various temperatures. They found that as the temperature increased from 293 K to 2373 K, Young’s modulus decreased from 397 GPa to 282 GPa. At temperatures below 0.5 *T*_m_ (where *T*_m_ is the melting point), Young’s modulus decreases linearly with increasing temperature; however, when the temperature exceeds 0.5 *T*_m_, an exponential decay term must be included to accurately describe the relationship between Young’s modulus and temperature. Nanoindentation measurements conducted by Cheng et al. [10] showed that at 573 K, the hardness and Young modulus of ZrC decreased by 27% and 10%, respectively, compared to their values at 298 K. Overall, due to the complexities involved in sample preparation and the challenge of maintaining stability at extreme temperatures [11], there is a scarcity of experimental data regarding the high-temperature mechanical properties of ZrC. Based on density functional theory (DFT) calculations, Zhang et al. [11] predicted the bulk modulus, shear modulus, Young modulus, and hardness of ZrC across a temperature range of 0 to 2500 K. On the other hand, the molecular dynamics (MD) method provides atomic-scale insights into the mechanical properties and internal structures of materials at different temperatures. Recently, Huang et al. [12] employed MD simulations to compare the tensile and shear behavior of ZrC at 300 K and 800 K using a machine-learned interatomic potential. However, there has been limited research on the nanoindentation behavior of ZrC using the MD method. Studies on nanoindentation in other carbide ceramic systems, such as SiC, have indicated that both the maximum indentation load and the yield load decrease as temperature increases [13,14]. Additionally, hardness decreases linearly with temperature [13,14], while Young’s modulus decreases exponentially [14]. As temperature increases, plastic deformation processes such as stacking faults (SFs), dislocations, and phase transformations are promoted, and new slip systems may be activated at elevated temperatures [13,14,15].

ZrC is typically non-stoichiometric. According to the Zr-C phase diagram, ZrC*_x_* (*x* = 0.49–1.00) adopts the rock-salt structure [16]. Numerous experiments have demonstrated that C vacancies significantly affect the mechanical, corrosion, and radiation properties of ZrC*_x_* ceramics [9,17,18,19,20,21,22,23,24,25,26,27,28]. In general, Young’s modulus and hardness tend to decrease as the C/Zr ratio decreases [9,23,25,27]. This relationship may be linear [27] or appear linear after correction [9,22]. However, some experiments have shown that mechanical properties do not vary monotonically with changes in the stoichiometric ratio. For instance, a decrease in the hardness of nearly stoichiometric ZrC has been observed, typically attributed to factors such as porosity [24,26], the presence of second phases [24], or sample grain orientation [22]. Recently, Huang et al. [12] found through MD simulations that this non-monotonic behavior arises from the competition between matrix softening and grain boundary strengthening due to C vacancies. Within a certain stoichiometric range, C vacancies can reduce stress concentration at neighboring Zr–C bonds, thereby enhancing the yield strength and strain at grain boundaries. Numerous DFT calculations [29,30,31,32,33] have been conducted to investigate the mechanical properties of ordered and disordered ZrC*_x_* at various stoichiometric ratios. Some DFT studies have also explored the impact of different vacancy configurations on mechanical properties. For instance, Zhang et al. [29] performed calculations on thirteen ZrC*_x_* structures and found that various mechanical parameters, such as Young’s modulus and hardness, decrease linearly with increasing C vacancy concentration, while showing little dependence on the vacancy configuration. In contrast, Xie et al. [31] demonstrated through their analysis of ten ZrC*_x_* structures that the relationship between hardness and C vacancy concentration is clearly nonlinear and is strongly influenced by the vacancy configuration. Overall, current research on the effects of stoichiometry ratios in ZrC*_x_* has primarily relied on DFT calculations. In other materials, such as Fe [34,35] and Cu [36], MD simulations have explored how vacancies influence the mechanical response during nanoindentation. It has been observed that dislocations tend to nucleate preferentially at vacancy sites [37]. For instance, in Fe, when the vacancy concentration is less than 0.1%, it has minimal influence on elastic deformation, and the yield strength decreases according to a power-law relationship as carbon concentration increases [35]. However, when the vacancy concentration exceeds 1%, its effect on the elastic deformation becomes considerable, and the elastic modulus decreases exponentially [34].

In addition, there are notable discrepancies in the hardness values obtained from experiments. For instance, the hardness of ZrC_1.0_ at room temperature ranges from 12.2 to 48.0 GPa, as reported by multiple studies [10,22,24,25,26,38]. One source of these discrepancies is the orientation preference of the sample [22]. Regarding the anisotropy of ZrC’s mechanical properties, experimental findings indicated that compression along the <111> direction is easier than along the <100> direction, and the (111) plane is up to 10 times softer than the (100) plane [39]. Furthermore, earlier research indicates that the nanoindentation hardness of ZrC (001) films is higher than that of the (111) plane [40]. DFT calculations have been conducted to compare the mechanical properties of the (100), (110), and (111) crystal planes in ZrC [11,32,41,42]. The results indicated that Young’s modulus is highest for the (100) plane and lowest for the (111) plane, and that Young’s modulus is positively correlated with the surface energy of the respective planes [41]. However, the findings for nickel suggested a different relationship: the surface with the densest atomic arrangement, which likely has lower surface energy, exhibits the highest modulus [43].

Previous experimental studies have shown that temperature [9,10], stoichiometry ratio [9,22,23,24,26,27], and crystal orientation [22] significantly influence the mechanical behavior of ZrC. However, there remains debate regarding the relationship between these variables and the resulting mechanical properties. To date, few studies have explored the dynamic response and corresponding microscopic mechanisms of this material at the atomic scale during mechanical loading. In this study, we simulate the nanoindentation process of ZrC using MD methods, based on a potential function developed in our previous work [44]. The effects of temperature, stoichiometry ratio, and the crystal plane of the sample are investigated. A comprehensive analysis of mechanical properties, defect structures, dislocation lengths, and distributions of stress and strain is conducted. The results provide a clearer understanding of the mechanical behavior of ZrC.

## 2. Model and Simulation Method

### 2.1. Simulation Model

**Figure 1:**
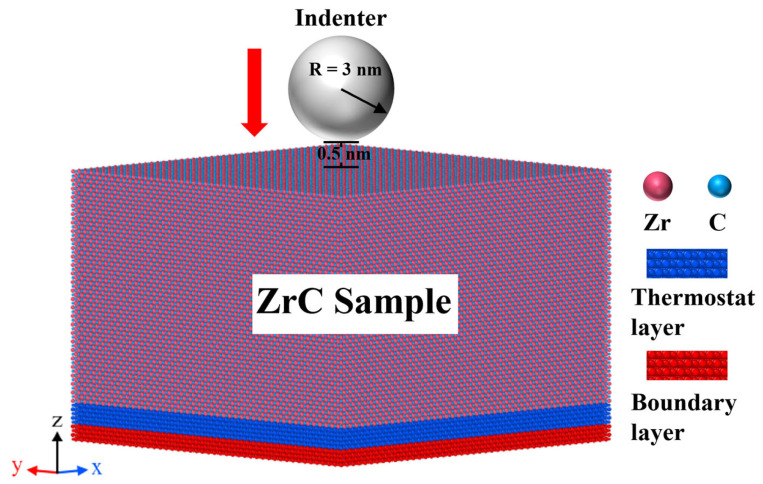
Schematic view of the simulation model.

Figure 1 shows a schematic diagram of the system used for nanoindentation simulations. The MD model consists of a virtual rigid spherical indenter and a single-crystal sample of stoichiometric ZrC or sub-stoichiometric ZrC*_x_* (*x* = 0.5–0.9). The sub-stoichiometric ZrC*_x_* samples, which contain disordered carbon vacancies, are constructed using the Special Quasi-Random Structures (SQS) method within the Alloy Theoretic Automated Toolkit (ATAT) software (version 3.36) [45]. The dimensions of the ZrC*_x_* (*x* = 0.5–1.0) samples are 21.19 nm × 21.19 nm × 15.07 nm in X, Y, and Z orientations, containing 388,800–518,400 atoms, depending on the composition. The sample surface is oriented in the (001) direction, with a spherical indenter of 3 nm radius located 0.5 nm above the sample. For stoichiometric ZrC, anisotropy effects are also considered, and the sample surfaces are oriented in [001], $[1\overset{-}{1}0]$, and $[1\overset{-}{1}1]$ directions. The coordinate systems and dimensions of stoichiometric ZrC samples are listed in Table 1. The sample consists of three parts: the bottom four layers are fixed to prevent the sample from shifting; the thermostat layer maintains the desired temperature of the sample; and in the Newtonian layer, the motion of the atoms follows Newton’s second law. The indenter is virtually realized by applying the force to atoms near the surface according to

(1) $$F\left(r\right)=\left\{\begin{array}{cc} -k{\left(R-r\right)}^{2} & r<R\\ 0 & r\geq R \end{array}\right.$$

where *k* is a force constant, which is set to 10 eV/Å, while *r* and *R* denote the distance between the center of the indenter and each atom and the indenter radius, respectively.

Prior to nanoindentation testing, the system is equilibrated at the desired temperature in the isothermal–isobaric (NPT) ensemble for 20 ps with a time step of 1 fs. Then, the indenter is pressed into the sample in the negative Z direction. Periodic boundary conditions are imposed in the X and Y directions, while free boundary conditions are used in the Z direction. The canonical (NVT) ensemble is applied to the thermostat layer to regulate atomic velocities according to the target temperature. Simultaneously, the microcanonical (NVE) ensemble is applied to the Newtonian layer. The indenter is performed under a constant speed of 20 m/s during both loading and unloading. Although this speed is higher than realistic conditions, it is widely accepted in MD simulations, and researchers have confirmed that it has little effect on the results [46]. As shown in Figure S1 and Table S1, reducing the indenter speed by half has a negligible effect on the mechanical behavior. The indentation depth is 3 nm. The temperatures are set to 10 K 300 K, 900 K, 1500 K, and 2100 K. All MD simulations are performed using the Large-scale Atomic/Molecular Massively Parallel Simulator (LAMMPS) code (version 29 October 2020) [47]. The simulation results are analyzed with the Open Visualization Tool (OVITO, version 3.12) [48]. Dislocations generated are identified and extracted using the Dislocation Extraction Algorithm (DXA) method. The centrosymmetric parameter (CSP) visualization technique is applied to analyze local plastic deformation. Although CSP was originally developed for body-centered cubic (BCC) and face-centered cubic (FCC) lattice structures, it can also be used for B1 (NaCl) structures by treating them as two identical FCC lattices.

### 2.2. Interatomic Potential

The reliability of mechanical responses in MD simulations depends on the accuracy of interatomic interaction potentials. Several interatomic interaction potentials have been developed for the Zr-C system. These include traditional empirical potentials such as the force-based many-body (MB) interatomic potential [49] developed by Li et al., as well as the second-nearest-neighbor modified embedded atom method (2NM-MEAM) potential [44] recently proposed by us. In addition, machine-learning-based potentials have been constructed, including the analytical bond-order potential (ABOP) [50], the spectral neighbor analysis potential (SNAP) [51], the moment tensor potential (MTP) [52], the deep learning potential (DLP) [12] and the deep potential (DP) [53]. The predicted lattice constants and mechanical constants of ZrC obtained from these potential functions are summarized in Table 2 and compared with DFT results and experimental data. It can be seen that the MEAM, MTP, DLP and DP provide a better description of the mechanical properties of ZrC. Furthermore, machine learning-based potential fields are typically several orders of magnitude less computationally efficient than empirical potential fields [54]; therefore, the MEAM potential field is used in this study to perform a series of nanoindentation simulations. The total energy of an atomic ensemble in the MEAM potential is expressed as follows:

(2) $$E_{i}=\sum_{i}\left\{F_{i}\left(\rho_{i}\right)+\frac{1}{2}\sum_{i\neq j}S_{ij}\phi_{ij}\left(r_{ij}\right)\right\}$$

where *F_i_* is the embedding energy for an atom *i* embedded in a background electron density *ρ_i_*, and *S_ij_* and *ϕ_ij_* (*r_ij_*) are the screening function and the pair interaction between atoms *i* and *j* with a distance *r_ij_*, respectively.

$$B=\left(C_{11}+2C_{12}\right)/3,\;G=\left(G_{V}+G_{R}\right)/2,\;G_{V}=\left(C_{11}-C_{12}+3C_{44}\right)/5,\;\nu =\left(3B-2G\right)/\left[2\left(3B+G\right)\right]$$

$$G_{R}=5\left(C_{11}-C_{12}\right)C_{44}/\left[4C_{44}+3(C_{11}-C_{12})\right],\;E=9BG/\left(3B+G\right),\;H_{V}=2{\left(G^{3}/B^{2}\right)}^{0.585}-3$$

### 2.3. Analysis Methodology

Several analytical methods are employed to study the nanoscale deformation behavior of ZrC crystals during nanoindentation. The von Mises stress is calculated to analyze shear-dependent deformation behavior, and the expression is as follows [61]:

(3) $$\sigma_{Mises}=\sqrt{\frac{6\sigma_{xy}^{2}+6\sigma_{yz}^{2}+6\sigma_{zx}^{2}+{\left(\sigma_{xx}-\sigma_{yy}\right)}^{2}+{\left(\sigma_{yy}-\sigma_{zz}\right)}^{2}+{\left(\sigma_{zz}-\sigma_{xx}\right)}^{2}}{2}}$$

where σ*_xx_*, σ*_yy_*, σ*_zz_*, σ*_xy_*, σ*_yz_* and σ*_zx_* are the six stress components for each atom. To quantify plastic deformation, atomic strain analysis is utilized based on the method proposed by Shimizu et al. [62]. The von Mises shear invariant can be calculated using the following formula:

(4) $$\eta_{Mises}=\sqrt{\frac{6\eta_{xy}^{2}+6\eta_{yz}^{2}+6\eta_{zx}^{2}+{\left(\eta_{xx}-\eta_{yy}\right)}^{2}+{\left(\eta_{yy}-\eta_{zz}\right)}^{2}+{\left(\eta_{zz}-\eta_{xx}\right)}^{2}}{6}}$$

Nanoindentation-tested hardness and Young’s modulus of the sample can be determined from the load–displacement curve using the Oliver–Pharr method [63]. The hardness is calculated based on the maximum load applied by the indenter and the projected contact area between the indenter and the material, as given by

(5) $$H=\frac{P_{max}}{A_{c}}$$

where *P_max_* is the maximum indentation load at the maximum indentation depth, while *A_c_* denotes the projected contact area and can be directly calculated with the formula below [64]:

(6) $$A_{c}=\pi \left(2R-h_{c}\right)h_{c}$$

where *R* is the radius of the spherical indenter, and *h_c_* is the projected contact depth between the indenter and the sample.

Young’s modulus can be calculated using the following equation:

(7) $$\frac{1-\nu^{2}}{E}=\frac{1}{E_{r}}-\frac{1-\nu_{indenter}^{2}}{E_{indenter}}$$

where *E_r_* represents the reduced elastic modulus, and *ν* and *ν_indenter_* are the Poisson ratios of the indenter and the sample, respectively. In this study, a rigid virtual indenter is used, and the value of *E_indenter_* is assumed to be infinite. Therefore, Equation (7) simplifies to

(8) $$E=E_{r}\times \left(1-\nu^{2}\right)$$

where the value of *ν* is set to 0.22 (see Table 2), derived from MD simulations of elastic constant testing using the MEAM potential function. *E_r_* can be determined using the following equation:

(9) $$E_{r}=\frac{1}{\beta }\frac{\sqrt{\pi }}{2}\frac{S}{\sqrt{A_{c}}}$$

where *S* is the initial slope at the top of the unloading curve, and *β* is a constant related to indenter shape. For a spherical indenter, *β* = 1.

## 3. Results and Discussion

### 3.1. Temperature Effect on Mechanical Properties of (001) Plane ZrC

**Figure 2:**
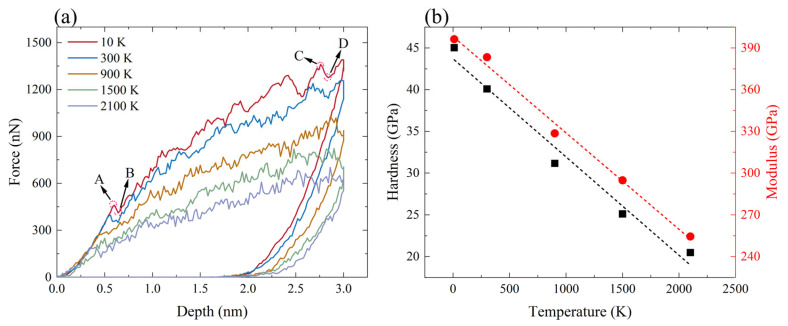
(**a**) Load–displacement curves and (**b**) hardness and Young’s modulus in (001) plane ZrC at different temperatures.

The temperature has a significant impact on the nanoindentation deformation characteristics of a material. Figure 2a displays the load–displacement curves for ZrC at various temperatures (ranging from 10 K to 2100 K). It is observed that the maximum load decreases as temperature increases, while the residual depth after unloading the indentation shows an overall increasing trend. Similar phenomena were observed in simulations of SiC [13,14], while the results for GaN indicated that the residual depth changed little with temperature [65]. The pop-in events in the load–displacement curves mean the transition from elastic to plastic deformation in the ZrC sample, as marked by point A in Figure 2a. The critical load at which pop-in occurs decreases with increasing temperature, suggesting that higher temperatures facilitate plastic deformation.

**Figure 3:**
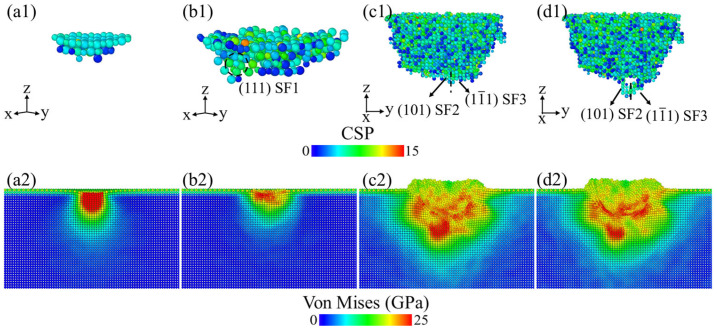
The configurations of defect atoms, with atom colors representing their CSP values, along with the von Mises stress distributions at several characteristic points in Figure 2 at 10 K. (**a1**,**a2**) at Point A; (**b1**,**b2**) at Point B; (**c1**,**c2**) at Point C; (**d1**,**d2**) at Point D.

The observed pop-in events and sudden load drops during indentation are presumably due to plastic deformation, such as the formation of dislocations and SFs. To clarify the plastic behavior, Figure 3 shows the defect microstructures and von Mises stress distributions at several characteristic points in Figure 2 at 10 K. To facilitate the observation of defects, surface atoms and atoms with CSP values < 1 are removed, leaving only the defect structures [66]. Between points A and B, the resolved shear stress reaches a critical value, leading to the nucleation of a (111) SF designated as SF1 beneath the indenter (Figure 3(b1)), accompanied by the release of internal stress (Figure 3(b2)). From points C to D, two SFs, referred to as SF2 and SF3, glide along the <110> direction on the (101) and $(1\overset{-}{1}1)$ planes, respectively (Figure 3(d1)). The nucleation and gliding of SFs result in stress release.

The hardness and Young modulus of the ZrC samples at different temperatures are calculated based on Equations (5) and (8), as shown in Figure 2b. As the temperature increases, hardness decreases roughly linearly. The maximum hardness occurs at 10 K, reaching 45.04 GPa, while the minimum hardness of 20.36 GPa is observed at 2100 K. At room temperature, the measured hardness is 40.09 GPa, with experimental values ranging from 12.2 to 48.0 GPa [10,22,24,25,26,38]. The hardness values obtained from MD simulations are higher than most experimentally measured results. This discrepancy is primarily due to size effects and defect effects [67,68]. Specifically, the dimensions of the simulated indenters are smaller than those used in experiments, leading to the “smaller is stronger” effect. In MD simulations, plastic deformation is driven by the formation and movement of a limited number of dislocations and SFs. In contrast, at the larger scales observed in experiments, plastic deformation results from the collective behavior of many dislocations and SFs. Additionally, the samples used in the simulations are ideal crystals without defects, whereas experimental samples contain various defects that can act as sources of dislocations, as well as pores and secondary phases (carbon) [22,24,26]. Young’s modulus also demonstrates an approximately linear decrease with increasing temperature. As the temperature rises from 10 K to 2100 K, Young’s modulus decreases from 396.28 GPa to 254.45 GPa. The value calculated at room temperature is 383.39 GPa, falling within the experimental range of 240.0 to 488.1 GPa [9,10,22,24,25,26,38,60].

**Figure 4:**
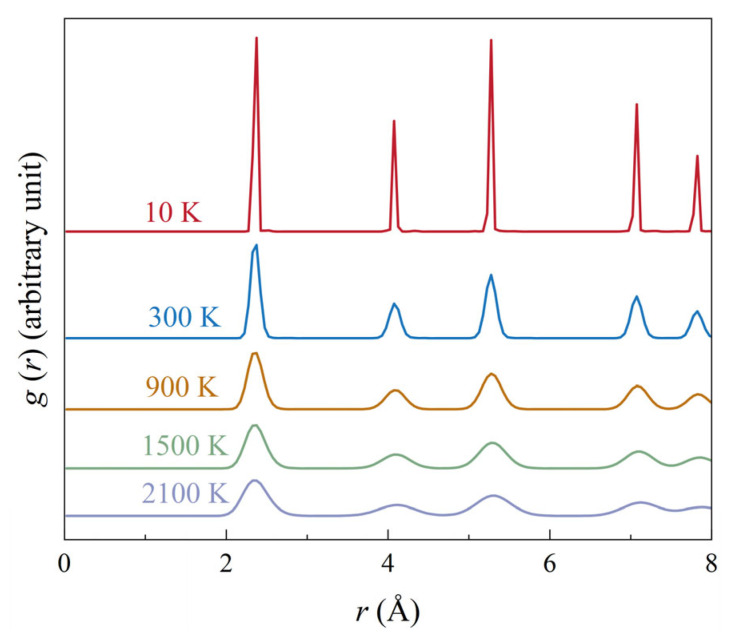
The RDF curves of Zr-C pairs for (001) plane ZrC before indentation at different temperatures.

The trend of decreasing hardness and Young’s modulus with increasing temperature is consistent with experimental results for the ZrC system [9,10]. The increase in thermal energy intensifies atomic vibrations within the material, leading to changes in interatomic distances and thereby weakening the covalent bonding energy between Zr and C atoms [14]. Figure 4 displays the radial distribution function (RDF) profiles of the Zr-C pair at different temperatures. The RDF provides information about the spatial arrangement of atoms around a central atom, revealing structural properties from the positions, widths, and intensities of the peaks. As the temperature rises, the peak value of *g*(*r*) decreases and broadens, indicating an increase in structural disorder. This increase in atomic thermal motion also leads to more pronounced fluctuations in the load–displacement curves as the temperature rises, as illustrated in Figure 2a.

**Figure 5:**
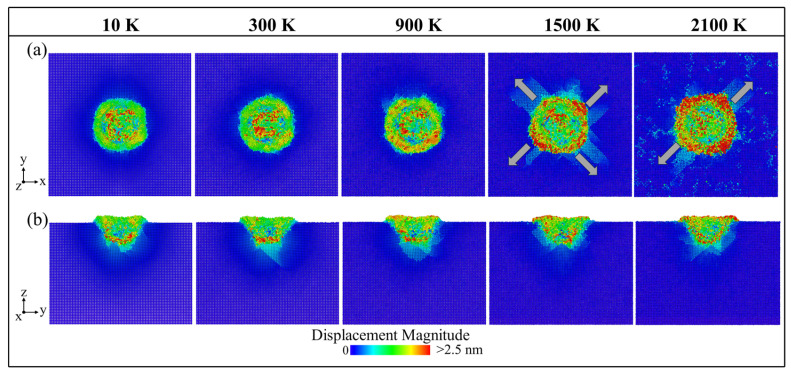
Atomic displacement magnitude distributions at maximum indentation depth of (001) plane ZrC at different temperatures: (**a**) top view; (**b**) cross-section view.

To analyze the deformation behavior at the atomic scale, Figure 5 illustrates atomic displacements at the maximum indentation depth across various temperatures. As the temperature increases, the area experiencing significant atomic displacements expands, indicating enhanced atomic movement. Beneath the indenter, atomic displacements primarily propagate in the <110> directions. When the temperature reaches 1500 K, the atoms tend to symmetrically expand along these directions on the surface. This suggests that elevated temperature can promote the development of subsurface dislocations. In crystals with a B1 structure, the <110> direction corresponds to the orientation of densely packed atoms. The smaller the atomic spacing between two atoms, the higher the probability of contact, facilitating slip along this direction.

**Figure 6:**
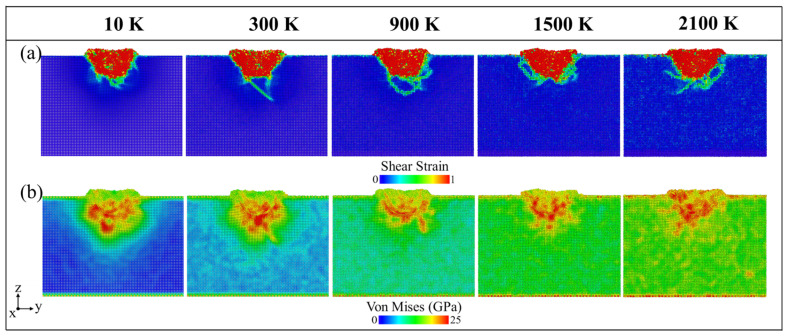
Distributions of the (**a**) shear strain and (**b**) von Mises stress at maximum indentation depth of (001) plane ZrC at different temperatures.

**Figure 7:**
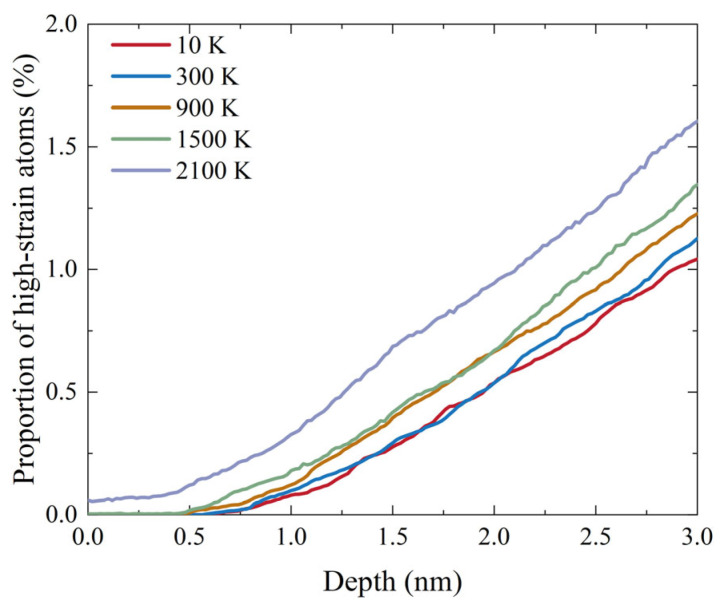
Proportion of high-strain (>0.5) atoms in (001) plane ZrC during nanoindentation loading at different temperatures.

Figure 6 shows the atomic shear strain and von Mises shear stress at the maximum indentation depth across various temperatures. The highest levels of atomic strain and stress are primarily found beneath the indenter. These elevated shear strains correspond directly to the areas of significant atomic displacements shown in Figure 5. As the temperature rises, both the strain values and their ranges continue to rise, indicating an enhanced capacity for local plastic deformation. Figure 7 presents the percentage of atoms experiencing high shear strain (>0.5) [69] during the nanoindentation loading at different temperatures. Changes in the slope of this curve are often associated with the initiation of pop-in events. As the temperature increases, a greater number of atoms undergo significant shear strain, making plastic deformation more likely. As a result, the imprint stress decreases.

**Figure 8:**
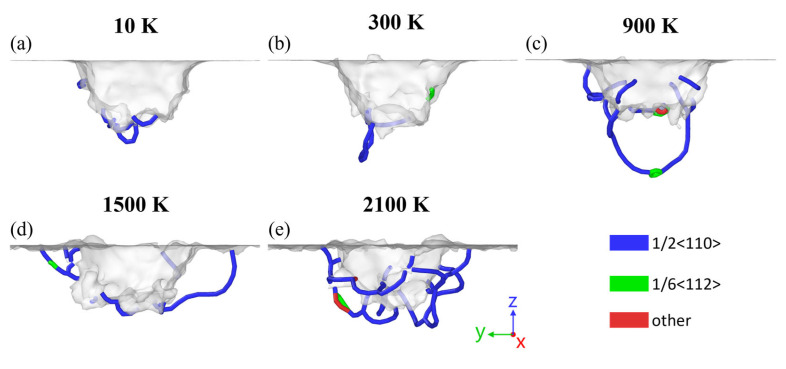
Snapshots of dislocations at a maximum indentation depth of (001) plane ZrC at different temperatures: (**a**) 10 K, (**b**) 300 K, (**c**) 900 K, (**d**) 1500 K and (**e**) 2100 K. Identification by DXA is achieved by removing C atoms.

**Figure 9:**
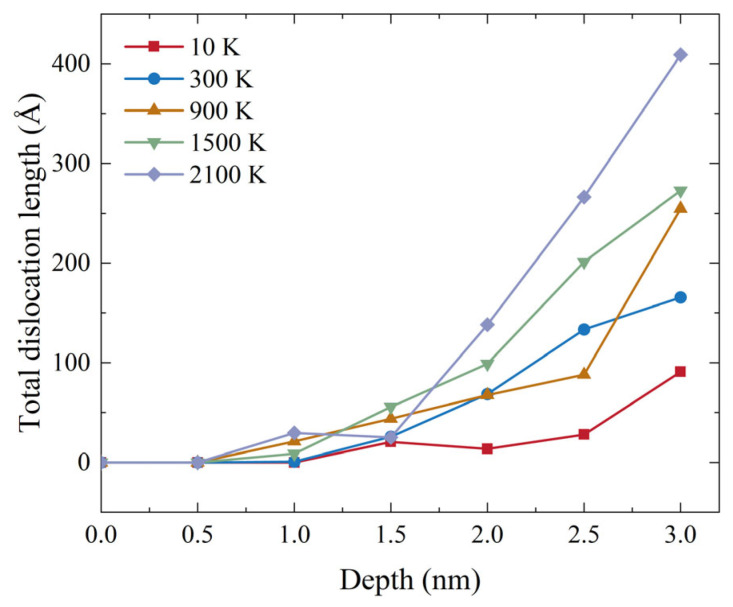
The curves of total dislocation length variation with indentation depth in (001) plane ZrC at different temperatures.

Figure 8 presents the morphologies of dislocations at the maximum indentation depth across various temperatures. Note that when considering only the elements Zr or C, the ZrC crystal adopts an i structure. The identification of the dislocations shown in Figure 6 is based solely on Zr atoms, similar to the method described in Ref. [70]. Dislocations with Burgers vectors of 1/2<110>, 1/6<112>, and other types that are not easily identifiable are observed. The predominant type of dislocation identified is the 1/2<110> dislocation, which aligns with experimental observation [71,72]. Figure 9 further illustrates the relationship between dislocation length and indentation depth at different temperatures. Dislocations begin to form at *h* = 1.5 nm below 900 K, and at *h* = 1.0 nm above 900 K. While there are some fluctuations, the overall trend shows that dislocation length increases with both indentation depth and temperature. Therefore, elevated temperatures promote the nucleation and propagation of dislocations, thereby enhancing the nanoscale plastic deformation capability of the ZrC sample.

### 3.2. Stoichiometric Ratio Effect on Mechanical Properties of (001) Plane ZrC_x_

**Figure 10:**
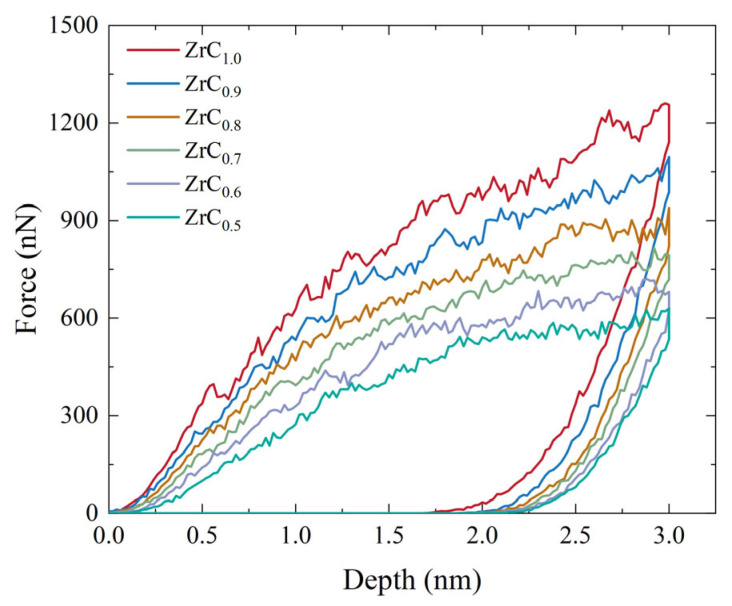
Load–displacement curves of (001) plane ZrC*_x_* with different C/Zr ratios at 300 K.

Figure 10 shows the load–displacement curves of ZrC*_x_* (*x* = 0.5–1.0) during nanoindentation at 300 K. The data reveal that the maximum load tends to decrease as the concentration of C vacancies increases, whereas the residual depth exhibits an overall increasing trend. Additionally, the slope of the initial elastic stage in the nanoindentation curve decreases with higher C vacancy concentration, indicating a reduction in the material’s resistance to elastic deformation. Previous computational studies have shown that a similar phenomenon in Fe occurs when the vacancy concentration exceeds 1% [34]. In contrast, when the vacancy concentration is below 0.1%, its impact on elastic deformation is minimal [35]. At different temperatures, the influence of the stoichiometric ratio of ZrC*_x_* on the behavior of the load–displacement curves remains consistent with the observations made at 300 K.

**Figure 11:**
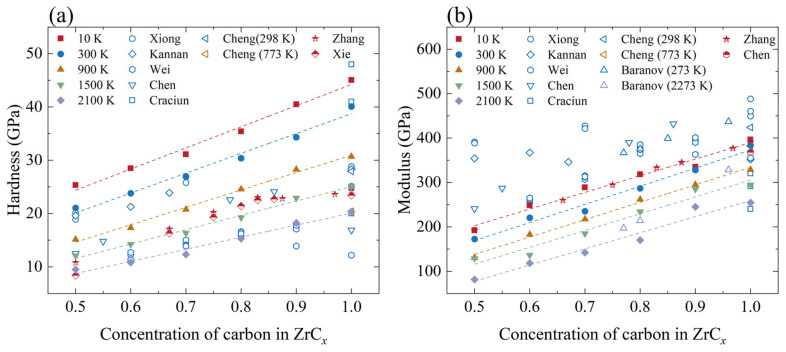
(**a**) Hardness and (**b**) Young’s modulus of (001) plane ZrC*_x_* with different C/Zr ratios across a temperature range from 10 K to 2100 K. Solid shapes represent the results from this MD simulations, hollow shapes represent experimental data [9,10,22,24,25,26,38,60], and semi-hollow shapes represent DFT results [29,31,73].

Figure 11a,b illustrate the calculated hardness and Young modulus of ZrC*_x_* (*x* = 0.5–1.0) with different C/Zr ratios across a temperature range from 10 K to 2100 K. The calculated results, along with experimental and DFT data from the literature, are presented in Tables S2 and S3. In Figure 11a, the hardness decreases linearly as the concentration of C vacancies increases at a constant temperature. At 300 K, when the C/Zr ratio decreases from 1.0 to 0.5, the hardness drops from 40.09 GPa to 21.04 GPa, a reduction of about 47.5%. The dashed lines in the figure represent the results of linear fitting. Similar to our simulation results, experimental studies by Kannan et al. [25] show that as the C/Zr ratio decreases from 1.0 to 0.5, the hardness decreases from 28.3 GPa to 19.6 GPa (a decrease of approximately 30.7%), exhibiting a monotonically decreasing trend. However, there are exceptions. A reduction in hardness was observed for nearly stoichiometric ZrC, which is typically attributed to factors such as pores [24,26], second phases [24], or preferred orientations in the samples [22]. Recently, Huang et al. [12] demonstrated through simulations that off-stoichiometry can have contrasting effects on bulk and grain boundary properties. The competition between bulk softening and grain boundary strengthening, driven by C vacancies, leads to non-monotonic variation in experimental outcomes. Since our model does not include grain boundaries, the influence of C vacancies on ZrC*_x_* hardness, as shown in Figure 11a, is more pronounced than what was observed in the experiments. Additionally, the slope of the dashed line decreases with rising temperature, indicating that the impact of C vacancies on the nanoindentation process diminishes. This phenomenon is attributed to the thermal softening of the substrate, which reduces the additional softening effect that the vacancies contribute.

Young’s modulus also exhibits a clear negative dependence on C vacancy concentration, as shown in Figure 11b. It is worth noting that at 10 K, as the C/Zr ratio decreases from 1.0 to 0.5, the simulated Young modulus decreased from 396.28 GPa to 192.09 GPa; this trend is in good agreement with the DFT results [29] (a decrease from 389.8 GPa to 192.2 GPa). Moreover, experiments have also revealed a linear relationship between Young’s modulus and the C/Zr ratio [27]. In this study, the slope of the linear fit at room temperature is 407, which falls between the experimental values of 379 and 505 [9,22]. This pattern of variation may differ depending on the material. For instance, in MD simulations of Fe, it was observed that as the vacancy concentration increased from 1% to 4%, the modulus decreased exponentially [34].

**Figure 12:**
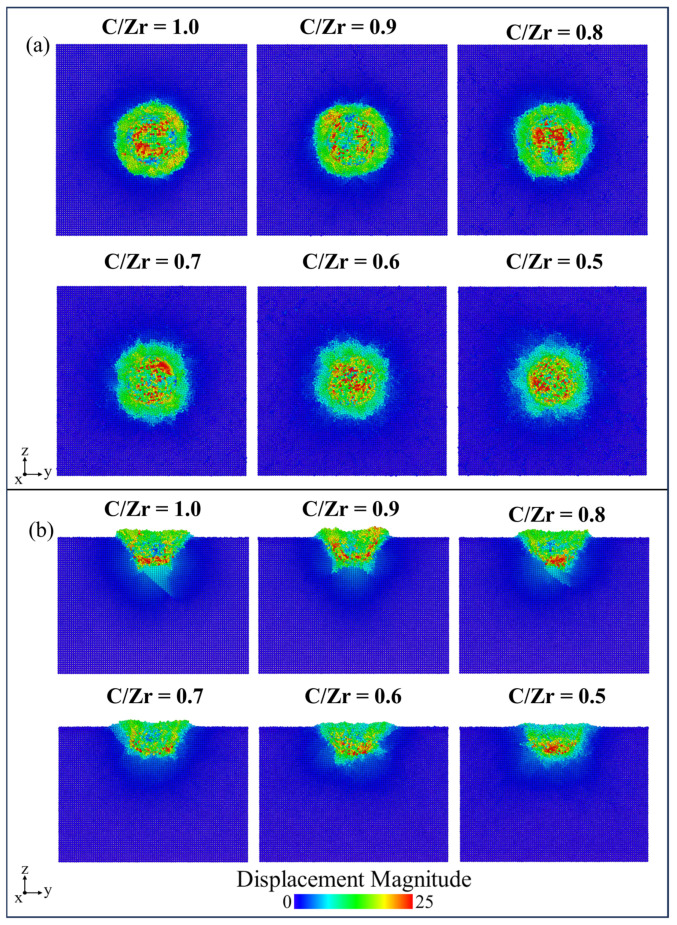
Atomic displacement magnitude distributions at maximum indentation depth of (001) plane ZrC*_x_* (*x* = 0.5–1.0) at 300 K: (**a**) top view; (**b**) cross-section view.

**Figure 13:**
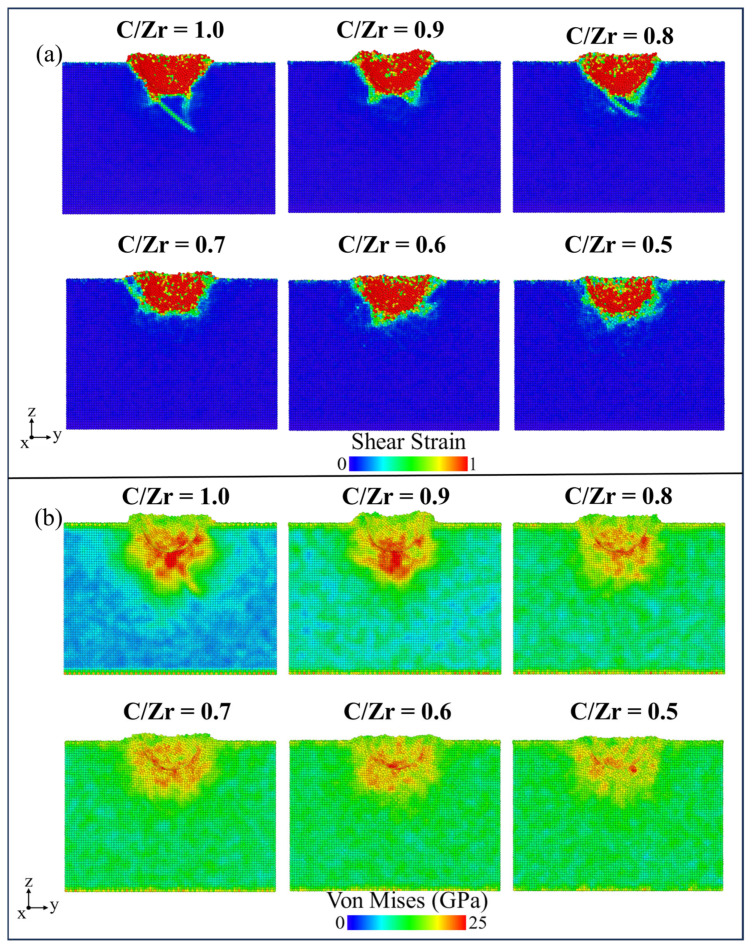
Distributions of the (**a**) shear strain and (**b**) von Mises stress at a maximum indentation depth of (001) plane ZrC*_x_* (*x* = 0.5–1.0) at 300 K.

**Figure 14:**
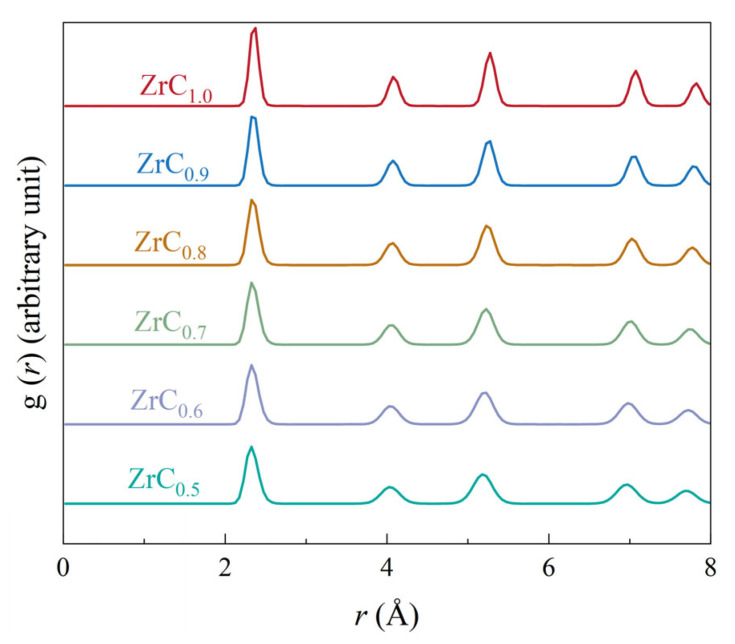
The RDF curves of Zr-C pairs for (001) plane ZrC*_x_* (*x* = 0.5–1.0) before indentation at 300 K.

To further investigate the deformation behavior of ZrC*_x_* samples, the distributions of atomic displacements, atomic shear strain, and von Mises stress are analyzed. Figure 12 illustrates the distribution of the atomic displacement magnitude in both the top and cross-section views of the ZrC*_x_* samples at the maximum indentation depth of 3 nm at 300 K. It is evident that the range of atoms that experienced displacement decreases as the C/Zr ratio decreases. For C/Zr ratios less than or equal to 0.7, atoms with significant displacement are primarily located directly beneath the indenter, indicating that they mainly move downward, while sliding in specific directions is suppressed. As depicted in Figure 13, in samples with a higher C/Zr ratio, stress becomes more concentrated beneath the indenter. A higher concentration of C vacancies leads to lattice distortion and weakens the binding between Zr and C atoms, making atoms more likely to move under load, thereby reducing stress accumulation and promoting plastic deformation. As the C/Zr ratio decreases, the shear bands beneath the indenter also become weaker. Figure 14 shows the RDF curves for Zr-C pairs at 300 K. As the C concentration increases, the peaks of the RDF curve decrease and shift to the left, indicating a contraction of the macroscopic lattice constant and a shortening of the Zr-C bond length, which in turn results in a decrease in the macroscopic hardness and Young’s modulus of ZrC*_x_*.

### 3.3. Crystal Plane Effect on Mechanical Properties of ZrC

**Figure 15:**
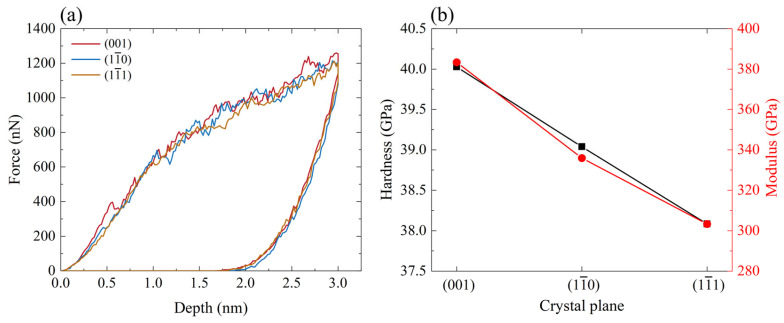
(**a**) Load–displacement curves and (**b**) hardness and Young’s modulus of stoichiometric ZrC at 300 K.

The hardness, Young modulus, and deformation behavior of a material are influenced by the direction of the applied external force. Figure 15a presents the load–depth curves obtained during the nanoindentation of stoichiometric ZrC for three different indentation planes. In the initial elastic stage, the slope for the (001) plane is higher than that of the $(1\overset{-}{1}0)$ and $(1\overset{-}{1}1)$ planes, indicating that the (001) plane exhibits the strongest resistance to elastic deformation. Obvious pop-in events are observed on the (001) and $(1\overset{-}{1}0)$ planes, corresponding to a transition from reversible elastic to irreversible plastic deformation. This directional movement of atoms during the process helps to dissipate stored elastic energy, resulting in a sudden drop in load [46]. In contrast, the pop-in phenomenon is less pronounced on the $(1\overset{-}{1}1)$ plane. Moreover, achieving the yield point on the (001) plane is the most challenging. This behavior aligns with the compression experiments on ZrC at room temperature, where sudden plastic deformation occurs on the (001) plane, while the load on the $(1\overset{-}{1}1)$ plane varies smoothly with displacement without any abrupt changes, and the yield strength of the $(1\overset{-}{1}1)$ plane is significantly lower than that of the (001) plane [39]. Furthermore, the (001) plane exhibits the highest indentation load at maximum indentation depth. All of these observations indicate that the (001) plane demonstrates the strongest resistance to plastic deformation.

Hardness and Young’s modulus calculated from the load–depth curves are shown in Figure 15b. The calculated results, along with experimental and DFT data from the literature, are presented in Table S4. The hardness values are 40.09 GPa for the (001) orientation, 39.04 GPa for the $(1\overset{-}{1}0)$ orientation, and 38.08 GPa for the $(1\overset{-}{1}1)$ orientation. The hardness values for these three planes fall within the experimentally reported range of 12.2 to 48 GPa for ZrC [10,22,24,25,26,38]. Additionally, the observation that (001)-oriented thin films exhibit higher hardness than (111)-oriented films aligns with previous experimental results [40]. Young’s modulus values for the (001), $(1\overset{-}{1}0)$, and $(1\overset{-}{1}1)$ crystal planes are 383.39 GPa, 335.93 GPa, and 303.31 GPa, respectively. It can be noted that the mechanical properties of the (001) plane of the ZrC film are superior to those of the other two indentation planes.

**Figure 16:**
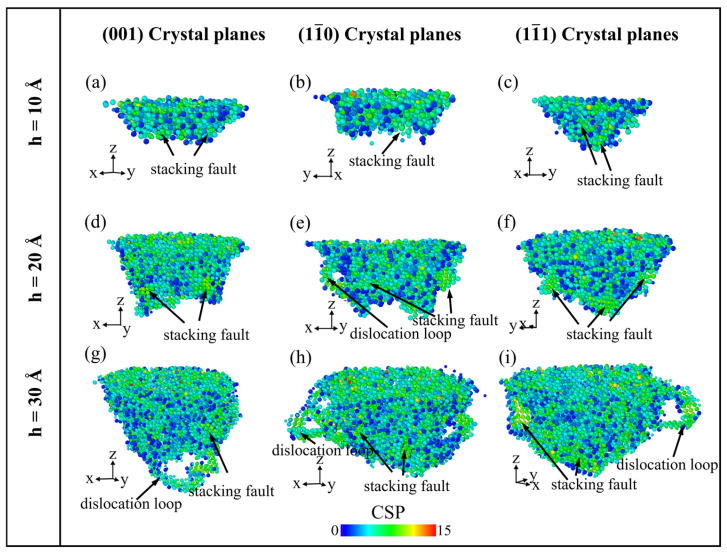
The distribution of defects in the (001) (**a**,**d**,**g**), $(1\overset{-}{1}0)$ (**b**,**e**,**h**), and $(1\overset{-}{1}1)$ (**c**,**f**,**i**) crystal orientations of stoichiometric ZrC under nanoindentation at various depths. The color of the atoms represents their CSP values.

Hardness is significantly influenced by plastic deformation, which is controlled by the generation and movement of dislocations. Figure 16 illustrates the distribution of CSP values during nanoindentation loading across various ZrC planes at different depths. To enhance the visibility of defects, surface atoms and those with CSP values less than 1 are removed, leaving only the defect structures. During nanoindentation, the nucleation and propagation of SFs and dislocations dominate plastic deformation. At shallower indentation depths, plastic deformation primarily occurs through the formation of SFs. In contrast, at greater indentation depths, the deformation process involves not only the formation of SFs but also dislocations. In terms of the spatial distribution of defects, those on the $(1\overset{-}{1}0)$ and $(1\overset{-}{1}1)$ planes are located around the tip of the indenter, while the defect structures on the (001) plane are primarily found directly beneath the indenter. At the same indentation depth, the $(1\overset{-}{1}0)$ and $(1\overset{-}{1}1)$ planes exhibit greater activity in SFs and dislocations compared to the (001) plane. As a result, the material can undergo plastic deformation more easily on the $(1\overset{-}{1}0)$ and $(1\overset{-}{1}1)$ planes, leading to lower hardness. Previous electron microscopy observations have indicated that compression forces applied on the (001) plane of ZrC activate the {110}<110> slip system. In contrast, compression on the $(1\overset{-}{1}1)$ plane may activate the {100}<110> slip system, which has a lower shear energy barrier and is, therefore, softer [39]. Our simulation results align with these experimental findings, showing a higher density of {100}<110> dislocations on the $(1\overset{-}{1}1)$ plane.

**Figure 17:**
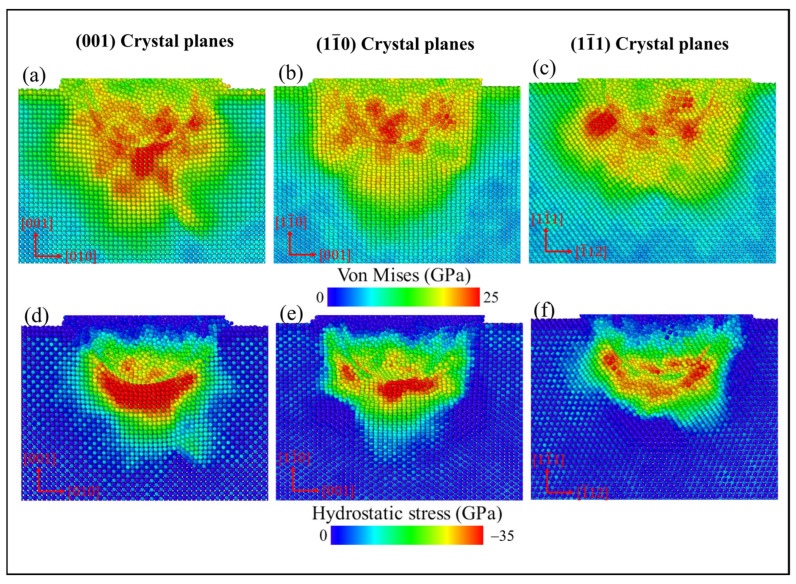
Distribution of the von Mises stress and hydrostatic stress on different crystal planes at maximum indentation depth: (**a**,**d**) for (001) crystal plane; (**b**,**e**) for $(1\overset{-}{1}0)$ crystal plane; (**c**,**f**) for $(1\overset{-}{1}1)$ crystal plane.

To illustrate the ease of dislocation slip across different crystal planes more clearly, Figure 17 presents the distribution of von Mises stress and hydrostatic stress at the maximum indentation depth. The von Mises stress provides an accurate assessment of the stress state during plastic deformation, while the hydrostatic stress is useful for evaluating whether the material is under compressive or tensile stress. The formula for calculating hydrostatic stress is $\sigma_{h}=\left(\sigma_{xx}+\sigma_{yy}+\sigma_{zz}\right)/3$. As shown in Figure 17, the region of von Mises stress distribution is broader than that of the hydrostatic stress distribution. This observation aligns with findings from studies on single-crystal silicon nanoindentation [74]. For the (001) plane, the high-stress region beneath the indenter is the largest, as clearly seen in the hydrostatic stress distribution. In contrast, the high-stress region is the smallest for the $(1\overset{-}{1}1)$ plane. The variations in stress magnitudes across different planes indicate how easily slip can be activated. A larger and more intense high-stress region means that a greater applied load is required to locally exceed the critical resolved shear stress needed for slip initiation. On the other hand, a smaller or weaker high-stress region suggests that slip can be activated more readily. Therefore, the (001) plane requires the highest stress concentration to induce plastic deformation, making slip activation most challenging here, resulting in the highest hardness. The plastic deformation occurs more easily on the $(1\overset{-}{1}1)$ crystal plane, which has the lowest hardness.

As shown in Figure 15b, Young’s moduli for the (001), $(1\overset{-}{1}0)$, and $(1\overset{-}{1}1)$ crystal orientations gradually decrease. This trend in Young’s modulus is consistent with the trends obtained from DFT calculations [11,32,41,42]. Previous computational studies of Ni metal and oxide crystals have shown that Young’s modulus of different crystallographic planes is correlated with surface energy (surface atomic density) [43,75]. Table 3 presents the calculated surface energies for the three planes using the MEAM potential function. Among these, the non-polar (001) plane exhibits the lowest surface energy due to its relatively high atomic density, whereas the polar $(1\overset{-}{1}1)$ plane shows the highest, in agreement with DFT calculations [76]. The negative correlation observed between Young’s modulus and surface energy can be explained by the fact that more stable surfaces exhibit greater bonding strength.

Meanwhile, the strong covalent bond in ZrC primarily arises from the Zr-C σ-bond formed by the hybridization of Zr-4d and C-2p orbitals. These bonds exhibit extremely high axial stiffness, making them very resistant to compress along the bond axis. When the loading direction deviates from the bond axis, bond angle distortion becomes dominant, which is relatively easier to accommodate. For the (001) plane, the bond directly beneath a surface atom is oriented perpendicular to the surface. This alignment means that the normal compressive direction coincides precisely with the axial direction of the σ-bond, resulting in maximum resistance and consequently the highest modulus. In contrast, for the $(1\overset{-}{1}0)$ and $(1\overset{-}{1}1)$ planes, the angles between the plane normal and the bond axis are 45° and 54.7°, respectively. Because of the greater proportion of bond angle distortion in these orientations, their moduli are lower.

**Figure 18:**
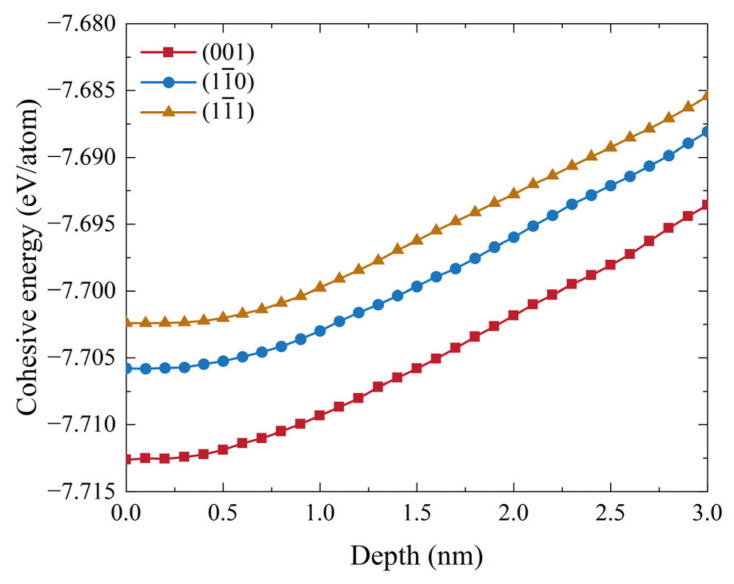
Cohesive energy curves between the substrate atoms of stoichiometric ZrC for the (001), $(1\overset{-}{1}0)$, and $(1\overset{-}{1}1)$ crystal planes.

The average atomic cohesive energy of samples with different indentation surfaces can be used to estimate Young’s modulus. Figure 18 illustrates the average atomic cohesive energy across three crystal planes. The *x*-axis represents indentation depth, while the *y*-axis shows the average cohesive energy of all atoms in the ZrC matrix, reflecting the average bonding strength between atoms. Throughout the indentation process, the cohesive energy of the (001) plane consistently remains the highest, while the $(1\overset{-}{1}1)$ crystal plane consistently exhibits the lowest cohesive energy. A higher cohesive energy corresponds to a higher Young modulus [64,77].

## 4. Conclusions

The effects of temperature, stoichiometric ratio, and crystal orientation on the nanoindentation response and mechanical properties of ZrC were studied by MD simulations. The impact of these factors was evaluated through loading force, atomic stress and strain, structural change and dislocation evolution. The main conclusions are as follows:

(1) Within the temperature range of 10 K to 2100 K, the nanoscale plastic deformation capability of the ZrC system increases significantly with temperature, as evidenced by decreases in critical pop-in load and maximum load and increases in residual indentation depth and atomic strain beneath the indenter. Both hardness and Young’s modulus decrease in a roughly linear manner with rising temperatures. With an increase in temperature from 10 K to 2100 K, the hardness of stoichiometric ZrC falls from 45.04 GPa to 20.36 GPa, and its Young modulus drops from 396.28 GPa to 254.45 GPa. The calculated Young modulus at 300 K is 383.39 GPa, which agrees well with previous experimental results. The plastic deformation is primarily governed by the nucleation and propagation of 1/2<110> type dislocations.
(2) For non-stoichiometric ZrC*_x_* (*x* = 0.5–1.0), both indentation hardness and Young’s modulus decrease linearly with increasing C vacancy concentration due to lattice distortion weakening Zr–C binding. This makes the atoms more likely to move under applied load, leading to reduced stress accumulation and increased plastic deformation. A reduction in the C/Zr ratio from 1.0 to 0.5 at 10 K leads to a decrease in Young’s modulus from 396.28 GPa to 192.09 GPa. Furthermore, the slope of the relationship between Young’s modulus and C concentration at room temperature aligns with experimental results.
(3) Both hardness and Young’s modulus for the (001), $(1\overset{-}{1}0)$, and $(1\overset{-}{1}1)$ crystal orientations gradually decrease. We observe that pop-in events are not pronounced on the $(1\overset{-}{1}1)$ crystal plane, as well as that the yield strength is the lowest on this plane. The magnitude relationships of Young’s moduli for the three planes are consistent with DFT results. Young’s modulus is negatively correlated with surface energy and with the angle between the plane normal and the direction of the Zr-C bond axis directly beneath the surface atom.

## Figures and Tables

**Table 1:** Coordinate systems and dimensions of stoichiometric ZrC samples.

Indented Plane	Crystal Orientations	Dimensions (Å)	Numbers of Atoms
ZrC (001)	X-[100], Y-[010], Z-[001]	211.98 × 211.97 × 150.74	518,400
ZrC $(1\overset{-}{1}0)$	X-[110], Y-[001], Z-$[1\overset{-}{1}0]$	213.01 × 211.97 × 153.10	529,920
ZrC $(1\overset{-}{1}1)$	X-[110], Y-$[\overset{-}{1}12]$, Z-$[1\overset{-}{1}1]$	213.01 × 219.06 × 154.90	554,496

**Table 2:** Calculated lattice constants *a* (Å), elastic constants *C_ij_* (GPa), bulk modulus *B* (GPa), shear modulus *G* (GPa), Young’s modulus *E* (GPa), Poisson’s ratio *ν*, and Vicker’s Hardness *H_V_* (GPa) for ZrC calculated using different potentials. The experimental and DFT results are also listed. “-” indicates the value is an unrealistic negative number.

	Experiments	DFT	MB [49]	MEAM [44]	ABOP [50]	SNAP [51]	MTP [52]	DLP [12]	DP [53]
a	4.688 [55], 4.69 [56], 4.694 [57]	4.707 [41], 4.67 [33], 4.71 [58]	4.698	4.707	4.712	4.78	4.713	4.691	4.71
C_11_	470 [59]	454.57 [41], 460.2 [33], 445.6 [58]	381.1	453.9	420.17	136.83	445.2	495	420
C_12_	100 [59]	105.83 [41], 118.1 [33], 103.5 [58]	121	107.79	106.45	240.52	154.55	95	99
C_44_	160 [59]	150.67 [41], 138.9 [33], 137.8 [58]	177.7	144.66	116.35	146.23	161.34	146	139
B	208 [60]	222.07 [41], 232.2 [33], 217.5 [58]	207.03	223.16	211.02	209	251.44	228	206
G	162 [60]	150.9 [33], 150.3 [58]	152.35	155.42	131.15	-	163.12	165.65	147.23
E	386 [60]	414.61 [41], 372.3 [33], 406.6 [58]	395.71	378.41	325.94	-	385.6	399.74	356.72
ν	0.1907 [60]	0.1888 [41], 0.189 [58]	0.24	0.22	0.23	0.71	0.26	0.21	0.19
H_V_	25.1 [24]	23.4 [31], 23.3 [41]	23.43	22.07	16.87	-	20.74	24.35	22.04

**Table 3:** The surface energies of ZrC (100), $(1\overset{-}{1}0)$, and $(1\overset{-}{1}1)$ calculated from MD simulations using the MEAM potential.

Crystal Plane	DFT [76] (J/m^2^)	MEAM (J/m^2^)
(001)	1.57, 2.93	3.25
$(1\overset{-}{1}0)$	3.21, 4.56	3.95
$(1\overset{-}{1}1)$	6.35, 5.28	4.26

## Data Availability

The original contributions presented in this study are included in the article and Supplementary Materials. Further inquiries can be directed to the corresponding author.

## Supplementary Materials

The following supporting information can be downloaded at https://www.mdpi.com/article/10.3390/ma19122581/s1. Figure S1: Load–displacement curves of ZrC_1.0_ and ZrC_0.6_ at different indentation velocities for (001) crystal plane at 300 K; Table S1: Hardness (GPa) and Young’s modulus (GPa) of ZrC_1.0_ and ZrC_0.6_ at different indentation velocities for (001) crystal plane at 300 K; Table S2: The hardness (GPa) of (001) plane ZrC_x_ with different C/Zr ratios across a temper-ature range from 10 K to 2100 K; Table S3: Young’s modulus (GPa) of (001) plane ZrC*_x_* with different C/Zr ratios across a temperature range from 10 K to 2100 K; Table S4: Hardness (GPa) and Young’s modulus (GPa) of the (001), $(1\overset{-}{1}0)$, and $(1\overset{-}{1}1)$ planes.
