# Supplementary material for: Effects of Temperature, Stoichiometric Ratio, and Crystal Orientation on the Nanoindentation Response of ZrC: A Molecular Dynamics Study
Source: Materials (Basel). 2026 Jun 15;19(12):2581. doi: 10.3390/ma19122581 (PMC13303983; doi:10.3390/ma19122581)
Supplement: Supplementary file 1 [file materials-19-02581-s001.zip › materials-4321888-supplementary.pdf]

materials

Supplementary material

Effects of Temperature, Stoichiometric Ratio, and Crystal Orientation on the Nanoindentation Response of ZrC: A Molecular Dynamics Study

Guiyu Liu, Hongya Zheng, Fugen Deng, Yulu Zhou * and Yifang Ouyang

State Key Laboratory of Featured Metal Materials and Life-Cycle Safety for Composite Structures, Guangxi Key Laboratory for Relativistic Astrophysics, School of Physical Science and Technology, Guangxi University, Nanning 530004, China; liugy@st.gxu.edu.cn (G.L.); zhenghongya@st.gxu.edu.cn (H.Z.); dfg@st.gxu.edu.cn (F.D.); ouyangyf@gxu.edu.cn (Y.O.)

* Correspondence: ylzhou@gxu.edu.cn

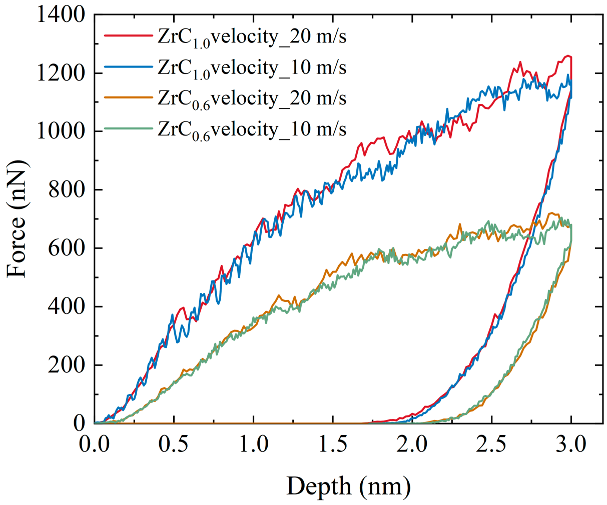

Received: 30 April 2026
Revised: 2 June 2026
Accepted: 9 June 2026
Published: date

Copyright: © 2026 by the authors. Submitted for possible open access publication under the terms and conditions of the Creative Commons Attribution (CC BY) license.

Figure S1. Load–displacement curves of $ZrC_{1.0}$ and $ZrC_{0.6}$ at different indentation velocities for (001) crystal plane at 300 K.

Table S1. Hardness (GPa) and Young's modulus (GPa) of $ZrC_{1.0}$ and $ZrC_{0.6}$ at different indentation velocities for (001) crystal plane at 300 K.

	Indentation velocity (m/s)	Hardness (GPa)	Young's modulus (GPa)
$ZrC_{1.0}$	10	39.04	373.46
	20	40.09	383.39
$ZrC_{0.6}$	10	23.29	212.84
	20	23.87	220.47

Table S2. The hardness (GPa) of (001) plane ZrC_x with different C/Zr ratios across a temperature range from 10 K to 2100 K.

C/Zr	Reference	10 K	300 K	900 K	1500 K	2100 K
1.0	Current work	45.04	40.09	30.67	25.12	20.36

	Experiments	—	28.8[1], 25.1[1], 19.1[1], 28.3[2], 12.1[3], 20[4], 41[4], 48[4], 27.9[5] (298 K), 16.9[6]	20.4[5] (773 K)	—	—
	DFT	23.6[7] ($ZrC_{0.97}$), 24.3[7], 23.4[8]	—	—	—	—
0.9	Current work	40.48	34.32	28.26	22.91	18.23
	Experiments	—	13.9[3], 17.1[3], 17.9[3], 17.8[3] 24.1[6] ($ZrC_{0.86}$)			
	DFT	22.8[7] ($ZrC_{0.875}$), 22.7[8] ($ZrC_{0.86}$)	—	—	—	—
0.8	Current work	35.39	30.37	24.56	19.24	15.29
	Experiments	—	16[3], 16.6[3], 16.5[3], 16.2[3] 22.6[6] ($ZrC_{0.78}$)	—	—	—
	DFT	23[7] ($ZrC_{0.83}$), 22.5[8] ($ZrC_{0.83}$), 21.4[8]	—	—	—	—
0.7	Current work	31.09	26.97	20.76	16.33	12.33
	Experiments	—	26.9[1], 25.8[1], 23.88[2] ($ZrC_{0.67}$), 15[3], 14.1[3], 13.9[3]			
	DFT	20.2[7] ($ZrC_{0.75}$), 19.3[8] ($ZrC_{0.75}$)	—	—	—	—
0.6	Current work	28.46	23.79	17.33	14.24	10.83
	Experiments	—	21.28[2], 12.7[3], 12.3[3], 11.3[3], 14.8[6] ($ZrC_{0.55}$)	—	—	—
	DFT	17.1[7] ($ZrC_{0.67}$), 16.3[8] ($ZrC_{0.67}$)	—	—	—	—
0.5	Current work	25.32	21.04	15.15	12.09	9.52
	Experiments	—	20.1[1], 18.9[1], 19.6[2], 12.5[6]	—	—	—
	DFT	10.8[7], 8.4[8]	—	—	—	—

Table S3. Young's modulus (GPa) of (001) plane ZrC_x with different C/Zr ratios across a temperature range from 10 K to 2100 K.

C/Zr	Reference	10 K	300 K	900 K	1500 K	2100 K
1.0	Current work	396.28	383.39	328.58	294.66	254.45
	Experiments	—	449.3[1], 488.1[1], 460.4[1], 352[2], 355[3], 240[4], 291[4], 320[4], 424[5] (298 K), 436.94[9] ($ZrC_{0.96}$, 273 K)	382[5] (773 K)	—	328.39[9] ($ZrC_{0.96}$, 2273 K)
	DFT	389.8[7], 368[10]	—	—	—	—
0.9	Current work	334.84	327.99	295.04	284.82	245.01
	Experiments	—	363[3], 397[3], 390[3], 401[3], 398.94[9] ($ZrC_{0.85}$, 273 K), 432[6] ($ZrC_{0.86}$)	—	—	—
	DFT	345[7] ($ZrC_{0.875}$)	—	—	—	—
0.8	Current work	317.96	286.61	261.77	234.44	170.15
	Experiments	—	365[3], 381[3], 385[3], 374[3], 375.76[9] ($ZrC_{0.8}$, 273 K) 390[6] ($ZrC_{0.78}$)	—	—	214.05[9] (2273 K)
	DFT	332.9[7] ($ZrC_{0.83}$)	—	—	—	—
0.7	Current work	288.84	235.11	217.21	184.8	142.15
	Experiments	—	427.4[1], 421.2[1], 346[2] ($ZrC_{0.67}$), 315[3], 307[3], 312[3], 313[3], 366.67[9] ($ZrC_{0.77}$, 273 K)	—	—	197.16[9] ($ZrC_{0.77}$, 2273 K)
	DFT	294.3[7] ($ZrC_{0.75}$)	—	—	—	—
0.6	Current work	248.15	220.47	182.84	136.55	118.48
	Experiments	—	367[2], 265[3], 255[3], 262[3], 287[6] ($ZrC_{0.55}$)	—	—	—
	DFT	259.7[7] ($ZrC_{0.67}$)	—	—	—	—
0.5	Current work	192.09	172.68	130.47	128.98	81.54
	Experiments	—	391.3[1], 388.4[1], 354[2], 241[6]	—	—	—
	DFT	192.2[7]	—	—	—	—

Table S4. Hardness (GPa) and Young's modulus (GPa) of the (001), $(1\bar{1}0)$, and $(1\bar{1}1)$ planes.

	**Reference**	**(001)**	**$(1\bar{1}0)$**	**$(1\bar{1}1)$**
Hardness	Current work	40.09	39.04	38.08
	Experiments	27.5[11]	—	17.6[11]
	DFT	—	—	—
Young's modulus	Current work	383.39	335.93	303.31
	Experiments	—	—	—
	DFT	368[10], 415[12], 383-414[13]	378[1]	366[12], 373[13]

Reference

1. Xiong, M.; Lu, Z.; Yan, S.; Chen, H.; Tao, X.; Ouyang, Y.; Li, Z.; Du, Y. Mechanical and thermal properties of densified ZrC_x (x=0.5, 0.7 and 1.0) ceramics. *J. Eur. Ceram. Soc.* **2024**, *44*, 1972-1982.
2. Kannan, R.; Venkateswarlu, K.; Rangaraj, L. Effect of nonstoichiometry on mechanical properties of reactive hot-pressed monolithic ZrC_x Ceramic. *Int. J. Appl. Ceram. Technol.* **2018**, *15*, 1366-1374.
3. Wei, B.; Chen, L.; Wang, Y.; Zhang, H.; Peng, S.; Ouyang, J.; Wang, D.; Zhou, Y. Densification, mechanical and thermal properties of ZrC_{1-x} ceramics fabricated by two-step reactive hot pressing of ZrC and ZrH_2 powders. *J. Eur. Ceram. Soc.* **2018**, *38*, 411-419.
4. Craciun, V.; McCumiskey, E.J.; Hanna, M.; Taylor, C.R. Very hard ZrC thin films grown by pulsed laser deposition. *J. Eur. Ceram. Soc.* **2013**, *33*, 2223-2226.
5. Cheng, E.J.; Li, Y.; Sakamoto, J.; Han, S.; Sun, H.; Noble, J.; Katsui, H.; Goto, T. Mechanical properties of individual phases of ZrB_2-ZrC eutectic composite measured by nanoindentation. *J. Eur. Ceram. Soc.* **2017**, *37*, 4223-4227.
6. Chen, L.; Lei, Y.; Zhang, J.; Wang, J. Synthesis and characterization of ZrC_x coatings with different stoichiometry. *Vacuum* **2022**, *202*, 111211.
7. Zhang, Y.; Liu, B.; Wang, J.; Wang, J. Theoretical investigations of the effects of ordered carbon vacancies in ZrC_{1-x} on phase stability and thermo-mechanical properties. *Acta Mater.* **2016**, *111*, 232-241.
8. Xie, C.; Oganov, A.R.; Li, D.; Debela, T.T.; Liu, N.; Dong, D.; Zeng, Q. Effects of carbon vacancies on the structures, mechanical properties, and chemical bonding of zirconium carbides: a first-principles study. *Phys. Chem. Chem. Phys.* **2016**, *18*, 12299-12306.
9. Baranov, V.M.; Knyazev, V.I.; Korostin, O.S.; Baranov, V.M.; Knyazev, V.I.; Korostin, O.S. The temperature dependence of the elastic constants of nonstoichiometric zirconium carbides. *Strength Mater.* **1973**, *5*, 1074-1077.
10. Chen, L.; Wang, Q.; Xiong, L.; Gong, H. Mechanical properties and point defects of MC (M=Ti, Zr) from first-principles calculation. *J. Alloys Compd.* **2018**, *747*, 972-977.
11. Woo, A.J.; Bourne, G.; Craciun, V.; Craciun, D.; Singh, R.K. Mechanical properties of ZrC thin films grown by pulsed laser deposition. *Journal of Optoelectronics and Advanced Materials* **2006**, *8*, 20.
12. Gusev, A.I. Effect of nonstoichiometry on anisotropy of elastic properties of disordered cubic zirconium carbide ZrC_y. *Int. J. Refract. Met. Hard Mater.* **2023**, *113*, 106192.
13. Khanzadeh, M.; Alahyarizadeh, G. A DFT study on pressure dependency of TiC and ZrC properties: Interconnecting elastic constants, thermodynamic, and mechanical properties. *Ceram. Int.* **2021**, *47*, 9990-10005.

Disclaimer/Publisher's Note: The statements, opinions and data contained in all publications are solely those of the individual author(s) and contributor(s) and not of MDPI and/or the editor(s). MDPI and/or the editor(s) disclaim responsibility for any injury to people or property resulting from any ideas, methods, instructions or products referred to in the content.
